# Innovative mHealth Intervention Providing Sustained Anticipatory Guidance (Zero Cavity): Design, Validation, User Perception and Effectiveness – A Randomized Controlled Trial Protocol

**DOI:** 10.64898/2026.09.11.26362881

**Published:** 2026-09-14

**Authors:** MS Muthu, Vineet Dhar, Ankita Saikia, S Vandana, R Harini, Kalpana Balakrishnan, Latha Nirmal, P Umapathy, John Davis

## Abstract

**Introduction:**

Early Childhood Caries (ECC) remains one of the most common chronic conditions in childhood, affecting over 600 million children globally. When untreated, it can lead to severe pain, abscesses, difficulty in chewing that contributes to malnutrition, impaired speech, disrupted sleep, low self-esteem, social withdrawal, and poor academic performance, all of which diminish a child’s overall quality of life. Although largely preventable, ECC remains highly prevalent because of delayed preventive care and low parental awareness. Effective health education needs to be consistent, accessible, and available whenever parents need it. Although current evidence indicates that anticipatory guidance is a proven approach, its long-term reinforcement through a mobile app has not yet been tested. This study aims to develop and evaluate the effectiveness of a mobile health (mHealth) application delivering Sustained Anticipatory Guidance (SAG) for the prevention of ECC.

**Methods and analysis:**

A two-arm parallel hospital-based randomized controlled trial will be conducted at Sri Ramachandra Institute of Higher Education & Research, Chennai, India. The study will enrol 540 mother-infant dyads from the departments of Pediatric Medicine and the Centre for Early Childhood Caries Research (CECCRe). Eligible dyads with infants aged 6–12 months in the predentate stage will be randomized to the “Zero-Cavity” mHealth app (intervention) or standard care (control). Baseline and semi-annual data on oral hygiene, fluoride exposure, and feeding/diet will be collected. Calibrated dentists will perform quarterly clinical exams using ICDAS II criteria, recording cavitated and non-cavitated lesions. For children at high risk or with enamel defects/incipient lesions, clinical photographs will be taken to support individualized preventive guidance. The primary outcome will be the effectiveness of the app in preventing ECC, while the secondary outcomes will include ECC incidence and prevalence.

**Discussion:**

Despite being largely preventable, ECC remains a major public health challenge, sustained by limited preventive access and inconsistent counselling, especially in LMICs. This trial evaluates the Zero Cavity app, believed to bridge this gap by providing sustained, age-appropriate anticipatory guidance beyond clinic settings on oral development, hygiene, diet/feeding, and fluoride use. The behavioural theory-driven approach is expected to enhance cultural relevance, usability, and behaviour change, with optimism that process evaluation will illuminate engagement and implementation. It is believed that this large, adequately powered, parallel-group RCT with intention-to-treat analysis will offer, with optimism, a scalable, low-cost model that will expand the reach and consistency of continuous preventive care in diverse urban and peri-urban populations.

**Trial registration:** CTRI/2026/04/107305

## Full Text Availability

The license terms selected by the author(s) for this preprint version do not permit archiving in PMC. The full text is available from the preprint server.

